# *d*-Limonene sensitizes docetaxel-induced cytotoxicity in human prostate cancer cells: Generation of reactive oxygen species and induction of apoptosis

**DOI:** 10.4103/1477-3163.51368

**Published:** 2009-05-21

**Authors:** Thangaiyan Rabi, Anupam Bishayee

**Affiliations:** Department of Pharmaceutical Sciences, Northeastern Ohio Universities Colleges of Medicine and Pharmacy, 4209 State Route 44, Rootstown, OH 44272, USA

**Keywords:** Apoptosis, *d*-limonene, docetaxel, DU-145, prostate cancer, reactive oxygen species

## Abstract

**Background:**

Clinical trials have shown that docetaxel combined with other novel agents can improve the survival of androgen-independent prostate cancer patients. *d*-Limonene, a non-nutrient dietary component, has been found to inhibit various cancer cell growths without toxicity. We sought to characterize whether a non-toxic dose of *d*-limonene may enhance tumor response to docetaxel in an *in vitro* model of metastatic prostate cancer.

**Materials and Methods:**

Human prostate carcinoma DU-145 and normal prostate epithelial PZ-HPV-7 cells were treated with various concentrations of *d*-limonene, docetaxel or a combination of both, and cell viability was determined by MTT assay. Intracellular reactive oxygen species (ROS), reduced glutathione (GSH) and caspase activity were measured. Apoptosis and apoptosis-related proteins were studied by enzyme-linked immunosorbent assay and Western blotting, respectively.

**Results:**

*d*-Limonene and docetaxel in combination significantly enhanced the cytotoxicity to DU-145 cells than PZ-HPV-7 cells. Exposure of DU-145 cells to a combined *d*-limonene and docetaxel resulted in higher ROS generation, depletion of GSH, accompanied by increased caspase activity than docetaxel alone. It also triggered a series of effects involving cytochrome *c*, cleavages of caspase-9, 3 and poly (ADP-ribose) polymerase, and a shift in Bad:Bcl-xL ratio in favor of apoptosis. Apoptotic effect was significantly blocked on pretreatment with *N*-acetylcystein, indicating that antitumor effect is initiated by ROS generation, and caspase cascades contribute to the cell death.

**Conclusion:**

Our results show, for the first time, that *d*-limonene enhanced the antitumor effect of docetaxel against prostate cancer cells without being toxic to normal prostate epithelial cells. The combined beneficial effect could be through the modulation of proteins involved in mitochondrial pathway of apoptosis. *d*-Limonene could be used as a potent non-toxic agent to improve the treatment outcome of hormone-refractory prostate cancer with docetaxel.

## INTRODUCTION

Prostate cancer is the second leading cause of cancer-related deaths in the United States.[1] Despite an initial efficacy of androgen-deprivation therapy, most patients with prostate cancer progress from androgen-dependent status to hormone-refractory prostate cancer (HRPC) for which there is no curative therapy.[2] Hence, the development of a novel and effective therapeutic strategy that could effectively inhibit hormone- and chemotherapy-refractory prostate cancer is urgently needed. Combination therapy utilizing multiple drugs is a common practice in the treatment of cancer. The promising clinical activity of docetaxel has prompted considerable interest in combining this drug with other antitumor agents, such as etoposide, cyclophosphamide, 5-fluorouracil, and doxorubicin.[3,4] A number of these docetaxel-containing combinations are currently undergoing clinical evaluations, and preliminary results appear to be encouraging. Although docetaxel chemotherapy has become the first-line standard for HRPC based on the results of two large randomized trials, prostate-specific antigen responses rarely exceed 50% and the median survival is less than 20 months, thus the use of chemotherapy in this disease remains a subject of active clinical investigation.[5] There are also several problems encountered during docetaxel treatment, including serious side effects in most of the patients.[6–8] The combination treatment is associated with a certain degree of dose-related toxicity. Thus, there exists an obvious need for the development of novel therapeutic strategies to improve efficacy and reduce side effects of docetaxel-based treatments for HRPC.

*d*-Limonene, a non-nutrient dietary component and major constituent in several citrus oils and a number of other essential oils, has been found to inhibit the growth of cancer cells without toxicity. *d*-Limonene, which comprises more than 90% of orange peel oil, has chemopreventive and chemotherapeutic activities against rodent mammary, liver and pancreatic tumors.[9–11] As a result, cancer chemotherapeutic activities of pharmacological preparations of *d*-limonene are under evaluation of a phase I/II therapeutic clinical trial.[12] *d*-Limonene, as a drug, is well tolerated in cancer patients at doses that may have clinical activity. A partial response in a breast cancer patient at a dose of 8 g/m^2^/day was maintained for 11 months, and three additional patients with colorectal carcinoma showed stabilization of disease for longer than 6 months on *d*-limonene at 0.5 or 1 g/m^2^/day.[13] The chemotherapeutic activity of *d*-limonene may be due to induction of apoptosis and redifferentiation concomitant with increased expression of mannose-6-phosphate/insulin-like growth factor II receptor and transforming growth factor B1.[14] *d*-Limonene and its *in vivo* plasma metabolites have been shown to be the inhibitors of protein isoprenylation of small G proteins, including p21 ras in rats.[15] In addition to selectively blocking the isoprenylation of small G proteins, *d*-limonene has also been shown to have additional cellular effects, including the inhibition of coenzyme Q synthesis, and it is also capable of causing the complete regression of the majority of advanced primary rat mammary carcinomas without significant toxicity.[16]

Based on the *in vitro* and *in vivo* experimental data, we hypothesize that a combined therapy of *d*-limonene and docetaxel may overcome the inhibition of apoptosis signaling pathways during the growth and survival of prostate cancer cells. In the current study, we have evaluated the *in vitro* antitumor potential of *d*-limonene in combination with docetaxel, using human prostate cancer DU-145 cells. We have also provided data in support of our hypothesis that a combination treatment causes greater antiproliferative and pro-apoptotic activities *in vitro*.

## MATERIALS AND METHODS

### Cell culture and reagents

The human prostate carcinoma DU-145 cells were kindly provided by Dr. Gail C. Fraizer (Kent State University, Kent, OH). DU-145 cells were cultured in RPMI 1640 medium (Invitrogen, Carlsbad, CA), supplemented with 1% penicillin/streptomycin and 5% fetal bovine serum, in a 5% CO_2_ atmosphere at 37°C. PZ-HPV-7 cells were maintained in keratinocyte serum-free medium (Life Technologies, Inc., Rockville, MD) supplemented with 5 ng/ml human recombinant epidermal growth factor and 5 *µ*g/ml bovine pituitary extract. The kit for the determination of glutathione (GSH) was purchased from Cayman Chemical (Ann Arbor, MI). The *N*-acetylcystein (NAC) and rotenone were purchased from Sigma-Aldrich (St. Louis, MO). 6-Carboxy-2,7-dichlorodihydrofluorescein diacetate (H_2_ DCFDA) was obtained from Molecular Probes (Eugene, OR). The antibodies against p21, p53, Bcl-2, cytochrome *c*, caspase-8, caspase-9, caspase-3, Bax, Bad, Bcl-xL and poly (ADP-ribose) polymerase (PARP) were purchased from Santa Cruz Biotechnology (Santa Cruz, CA). *d*-Limonene was received as a generous gift from Florida Chemical Company, Inc. (Winter Haven, FL). Docetaxel (purchased from LC Laboratories, Woburn, MA) was dissolved in dimethyl sulfoxide (DMSO) to prepare 1 mM stock solution and aliquots were stored at −80°C. Stock solutions were diluted to the desired final concentration with medium just before use.

### Cell growth inhibition by MTT assay

DU-145 and PZ-HPV-7 cells were seeded at a density of 5 × 10^3^ cells per well in 96-well microtiter culture plates. After overnight incubation, the medium was removed and replaced with a fresh medium containing various concentrations of *d*-limonene, docetaxel or a combination of both, and cell viability was determined by measuring the absorbance of 3-(4,5-dimethylthiazol-2-yl)-2,5-diphenyltetrasolium bromide dye (Sigma-Aldrich, St. Louis, MO) for living cells, as described earlier.[17] The concentration necessary to inhibit 50% of cell population (IC_50_) was determined by interpolation from dose-response curves.

### Measurement of reactive oxygen species (ROS)

Intracellular ROS generation was measured by the detection of O_2_ and H_2_O_2_.[18] Briefly, 5 × 10^5^ cells were plated in 60-mm dishes, allowed to attach overnight, and exposed to different concentrations of drugs alone or in combination for specified time intervals. The cells were stained with 5 *µ*M H_2_ DCFDA for 30 min at 37°C. The cells were collected, and the DCF fluorescence intensity proportional to the amount of intracellular ROS was measured using a microplate fluorometer at an emission wavelength of 538 nm and an excitation wavelength of 485 nm. ROS level was expressed as the fluorescence of the treated samples compared to the fluorescence of the control samples [(fluorescence of treated cells/fluorescence of control cells) × 100]. In separate experiments, cells were pretreated with NAC prior to drug exposure and analysis of ROS generation.

### GSH assay

The effect of drugs on the intracellular level of GSH was determined by using a kit from Cayman Chemical. Briefly, DU-145 cells were exposed to drugs for the specified time period at 37°C. The cells were collected, washed with phosphate-buffered saline (PBS), resuspended in PBS, and counted. An equal number of cells from each treatment group were used for the determination of GSH according to the manufacturer's instructions.

### Apoptosis detection by enzyme-linked immunosorbent assay (ELISA)

The cell apoptosis ELISA detection kit (Roche, Indianapolis, IN) was used to detect apoptosis following treatments with drugs alone and in combination according to manufacturer's protocol. In short, DU-145 cells were treated with docetaxel and *d*-limonene alone or in combination for 0, 6, 12, 24 and 48 h, after which the media was aspirated, the cells were washed with cold PBS and incubated on ice for 30 min in cell lysis buffer. The cells were then scraped and the lysate was collected in a microcentrifuge tube and vortexed to break up the cell aggregates. The lysate was cleared by centrifugation at 5,000 rpm for 10 min at 4°C and the supernatant was stored at −80°C. Protein concentration was determined by DC Bio-Rad protein assay kits (Bio-Rad Laboratories, Hercules, CA). The cell lysate with 50 *µ*g protein was added to the lysis buffer provided with the kit. The sample was then pipetted on a streptavidin-coated 96-well microtiter plate to which immunoreagent mix was added and incubated for 2 h at room temperature with continuous shaking at 500 rpm. The wells were then washed with washing buffer, and color was developed by addition of substrate solution, which was read at 405 nm against the blank, or at reference wavelength of 490 nm after 10-15 min. The enrichment factor (indicative of apoptosis) was calculated by dividing the absorbance of the sample (A405 nm) by the absorbance of the controls without treatment (A490 nm).

### Caspase activity detection assay

The M30 CytoDeath apoptosis detection kit (Roche, Mannheim, Germany) was used for the detection of apoptotic cells. Briefly, cells were grown in cover glasses and then treated with drugs alone or combination for 0, 6, 12, 24 and 48 h. The cells were then washed with cold PBS, fixed in ice-cold pure methanol at −20°C for 30 min and washed twice with washing buffer. The washing buffer was removed and the cells were incubated with M30 CytoDeath fluorescein antibody working solution for 1 h at room temperature. The apoptotic cells stained with green fluorescence were washed with washing buffer twice and examined under a fluorescence microscope according to the vendor's protocol.

### Western blot analysis

To determine the levels of protein expression in DU-145 cells, we prepared cell lysates, cytosolic fractions and fractionated them using sodium dodecyl sulfate-polyacrylamide gel electrophoresis. After electrophoresis, the proteins were electrotransferred to nitrocellulose membrane, blotted with each antibody, and detected using enhanced chemiluminescence reagent (Amersham, Piscataway, NJ).

### Statistical analyses

Data are expressed as means ± standard errors (SE). Analysis of data was performed using the Student's *t*-test, and a *P* value less than 0.05 was considered to be statistically significant.

## RESULTS

### Effect of docetaxel and *d*-limonene alone or in combination on cell growth

**Figure 1:**
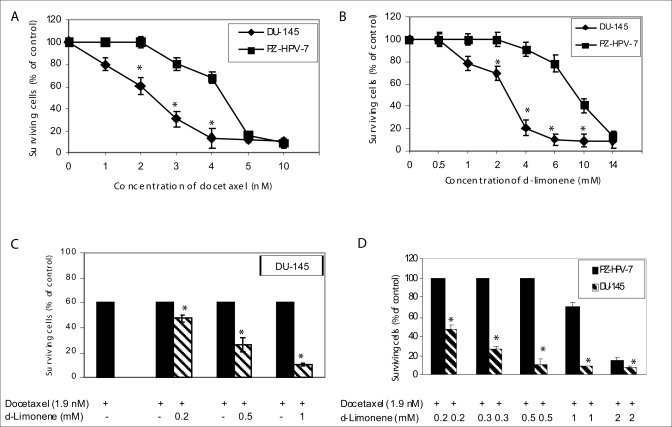
Effect of docetaxel and *d*-limonene on the growth of prostate cancer DU-145 and prostate epithelial PZ-HPV-7 cells by MTT assay. (A) Cells were either untreated or treated with increasing concentration of docetaxel or (B) *d*-limonene; (C) and (D) Cells were treated with either docetaxel alone or a combination of docetaxel and *d*-limonene for 48 h and then analyzed by MTT assay as described under Materials and Methods. Data represent the mean ± SE of triplicate determination. *P < 0.05 compared with corresponding control group.

Treatment with increasing concentrations of docetaxel or *d*-limonene inhibited the proliferation of DU- 145 cells in a dose-dependent manner [Figures 1A and B]. The IC50 values of docetaxel and *d*-limonene were 2.4 nM and 2.8 mM respectively. Both docetaxel and *d*-limonene killed prostate cancer DU-145 cells more effectively than the normal epithelial prostate PZ-HPV-7 cells with IC_50_ values of 4.4 nM and 9.4 mM respectively). The low IC_40_ concentration (1.9 nM) derived from the dose-response curve for docetaxel [Figure 1A] and concentrations of 0.2-2.0 mM for *d*-limonene were used to evaluate possible chemosensitizing effects in subsequent experiments. *d*-Limonene dose-dependently resulted in an enhancement of docetaxel-induced cytotoxicity in DU-145 cells [Figure 1C]. Combination of docetaxol and *d*-limonene caused greater cell proliferation inhibition of DU-145 cells than normal prostate epithelial PZ-HPV-7 cells [Figure 1D].

### Effect of docetaxel alone or in combination with *d*-limonene on ROS generation

**Figure 2:**
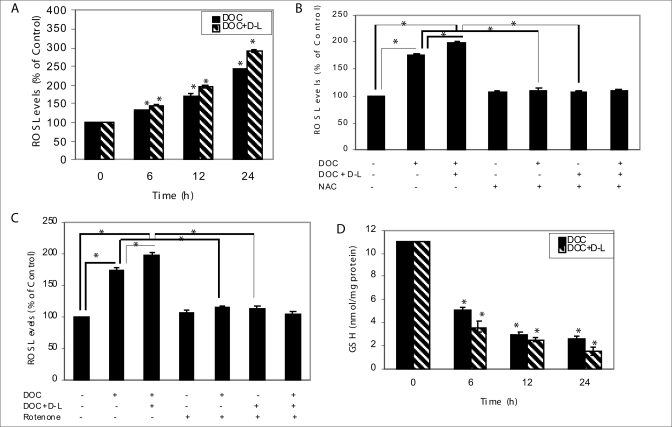
Docetaxel treatment alone or together with *d*-limonene caused ROS generation in DU-145 cells. (A) High DCF fluorescence in DU-145 cells following docetaxel treatment in combination with *d*-limonene for the indicated times in comparison to docetaxel alone. Data represent the mean ± SE of triplicate determination. *P < 0.05 compared with corresponding control. (B) DU-145 cells were pretreated with 10 mM NAC for 2 h and either left alone or exposed to 1.9 nM docetaxel alone or in combination with 0.2 mM *d*-limonene for 12 h. NAC pretreatment protected against docetaxel- and docetaxel plus *d*-limonene-induced ROS generation in DU-145 cells. Data represent the mean ± SE of triplicate determination. *Significantly different between the indicated groups (*P* < 0.05). (C) DU-145 cells were pretreated with 200 nM rotenone for 30 min and either left alone or treated with docetaxel only or in combination with *d*-limonene for 12 h and processed for analysis of ROS generation. Docetaxel and *d*-limonene combined treatment-mediated increase in DCF fluorescence was significantly inhibited on pretreatment with rotenone. Data represent the mean ± SE of triplicate determination. *Significantly different between the indicated groups (*P* < 0.05). (D) GSH levels in DU-145 cells treated with 1.9 nM docetaxel alone or together with 0.2 mM *d*-limonene for the indicated time periods. Data represent the mean ± SE of triplicate determination. *Significantly different between the indicated groups (*P* < 0.05). DOC, docetaxel; D-L, *d*-limonene; NAC, *N*-acetylcystein.

We next examined involvement of ROS in a single or combination treatment-induced cell death of prostate cancer cells. Treatment of docetaxel alone (1.9 nM) or in combination with *d*-limonene (0.2 mM) produced elevated levels of ROS in a time-dependent manner [Figure 2A]. ROS levels were increased after the combination treatment of docetaxel and *d*-limonene in DU-145 cells, and the increase in ROS level was effectively blocked by antioxidant NAC at a concentration of 10 mM [Figure 2B]. Antioxidant NAC efficiently suppressed docetaxel- and combination treatment-induced ROS generation suggesting that ROS might be an important factor to determine combination treatment-related sensitivity in human prostate cancer DU-145 cells. However, 10 mM NAC failed to affect the basal levels of ROS in DU-145 cells. These results suggest that NAC activity in DU-145 cells could be dependent as well as independent of its antioxidant functions. In addition, we found that pretreatment of rotenone (200 nM), a mitochondrial ROS blocker, completely attenuated the combination treatment-induced ROS generation in DU-145 cells [Figure 2C], suggesting that ROS generation in response to the combination treatment is mitochondria-dependent. We speculated that docetaxel together with *d*-limonene might cause GSH depletion to exacerbate oxidative stress. We therefore determined the effect of a combination treatment on intracellular levels of GSH, and the results are shown in Figure 2D. Treatment of DU-145 cells with docetaxel together with *d*-limonene for 6, 12 and 24 h caused a rapid decline in the level of GSH by about 67.3, 77.3 and 86.4%, respectively, compared with control at zero-time. Docetaxel treatment alone caused a decrease in the level of GSH by about 54.5, 72.7 and 76.4% for the same time-points. These observations led us to conclude that ROS generation probably involves both a non-mitochondrial mechanism and a mitochondria-mediated phenomenon.

### Effect of docetaxel alone or in combination with *d*-limonene on cytoplasmic histone-associated DNA-fragmentation in DU-145 cells

**Figure 3:**
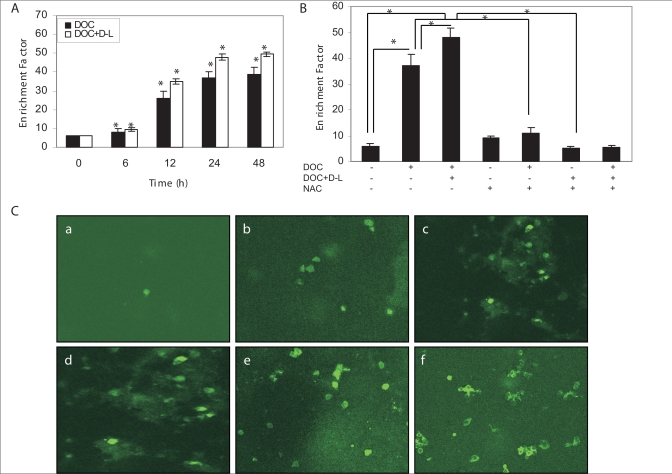
Sensitization of prostate cancer DU-145 cells to docetaxel-induced apoptosis as determined by histone-DNA ELISA after 0, 6, 12, 24 and 48 h treatment with docetaxel (1.9 nM) alone, or the combination of docetaxel and *d*-limonene (0.2 mM). (A) Increased apoptotic response as evidenced from higher enrichment factors in combination treatment group relative to docetaxel-treated group. Data are mean ± SE (*n* = 3). *P < 0.05 compared with corresponding control. (B) DU-145 cells were pretreated with 10 mM NAC for 2 h and either left alone or exposed to 1.9 nM docetaxel alone or in combination with 0.2 mM *d*-limonene for 12 h. NAC pretreatment protected against docetaxel and docetaxel plus *d*-limonene-induced apoptosis in DU-145 cells. Data are mean ± SE (*n* = 3). *Significantly different between the indicated groups (*P* < 0.05). (C) M30 antibody stained (a) control cells, (b) docetaxel plus *d*-limonene-treated cells for 6 h, (c) 12 h, (d) 24 h, (e) 48 h, and (f) docetaxel-treated cells for 48 h for assessing caspases activation as measured by intense staining of fluorescein. DOC, docetaxel; D-L, *d*-limonene; NAC, *N*-acetylcystein.

Apoptosis was studied by measuring the cytoplasmic histone-associated DNA fragments (mono- and oligo-nucleosomes) by ELISA. Treatment of DU-145 cells with 1.9 nM docetaxel for 6, 12, 24 and 48 h induced significant cytoplasmic histone-associated DNA-fragmentation and related enrichment factors were 8, 26, 37 and 39 respectively [Figure 3A]. These results show that docetaxel induces apoptotic cell death in DU-145 cells in time-dependent manner. We further examined whether docetaxel treatment in combination with *d*-limonene had a sensitizing effect on cell death in DU-145 cells. As shown in Figure 3A, the combination treatment synergistically enhanced the apoptotic cell death in the same manner as observed for the docetaxel alone. To further examine the direct relationship between the increased intracellular ROS level and mitochondrial activation-mediated cell death pathway, cells were pretreated with NAC before the single or combination treatment. Pretreatment of NAC significantly attenuated the combination treatment-induced apoptotic cell death, suggesting that ROS plays a crucial role in *d*-limonene-mediated enhancement of docetaxel-induced cell death [Figure 3B].

### Effect of docetaxel alone or in combination with *d*-limonene on caspase activity in DU-145 cells

To analyze apoptotic activity, the expression of the specific caspase cleavage site within cytokeratin 18 was assessed using the fluorescein-conjugated mouse monoclonal antibody (clone M30). In contrast to DMSO control group, docetaxel plus *d*-limonene treatment displayed intensive fluorescein staining between 6 and 48 h. Representative photographs of fluorescein binding to apoptotic cells after specific treatments are shown in Figure 3C.

### Effect of docetaxel and *d*-limonene combination treatment on the protein expression of p21, p53, Bad, Bax, Bcl-2, Bcl-xL, cytochrome c release, caspases and PARP

**Figure 4:**
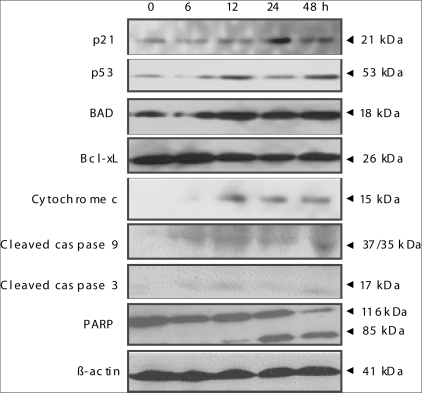
A combined treatment of docetaxel and *d*-limonene increased p21, p53, Bad, cytochrome *c* and decreased Bcl-xL protein levels and cleavage of caspase-9, caspase-3 and PARP during 0-48 h. Immunoblotting using cytosolic fraction (for cytochrome *c*) and cell lysates (for all other proteins) from DU-145 cells treated with 1.9 nM docetaxel together with 0.2 mM *d*-limonene for the indicated time periods. The blots were stripped and reprobed with anti-actin antibody to ensure equal loading.

The effects on the expressions of Bax, Bcl-2, Bad, Bcl-xL, caspases and PARP proteins were analyzed in a time-dependent manner to explain the biochemical mechanisms of the enhanced effect of *d*-limonene on docetaxel activity for DU-145 cells. To determine whether the mitochondrial pathway is involved in induction of the apoptotic cell death observed after combination treatment, we examined changes in cytochrome *c* release from the mitochondria into the cytosol after combination treatment. While we did not observe cytochrome *c* expression in the cytosol of untreated cells, the level of the cytosolic cytochrome *c* was markedly increased over time in the cells subjected to combined treatment [Figure 4]. Cleavage of caspase-9 and caspase-3, up regulation of p21, and Bad, cleavage of PARP and down regulation of Bcl-xL were observed in a time-dependent fashion for both drugs, when combined [Figure 4]. However, we were unable to detect caspase-8 cleavage, Bax up regulation and Bcl-2 down regulation (data not shown). Wild-type p53 provides a protective effect against neoplastic changes and tumor growth[19] and the p53 tumor suppressor gene is mutated in DU-145 cells.[20] Treatment of DU-145 cells with *d*-limonene and docetaxel combination resulted in increase in the expression of p53 protein [Figure 4]. Since p53-mediated suppression of transformation is dependent on the phosphorylation of p53 at Ser 15 residue,[21] we determined whether p53 is phosphorylated at Ser 15 in DU-145 cells after treatment with docetaxel and *d*-limonene combination. For this purpose, we used a phospho-specific antibody against p53 at Ser 15. Our Western blot analysis revealed that the level of phosphorylation of p53 increased between 12-48 h after exposure to docetaxel and *d*-limonene combination. The combined treatment of *d*-limonene and docetaxel did not alter the parameters related to the mitochondrial pathway of apoptosis in normal epithelial prostate PZ-HPV-7 cells under the similar experimental conditions (data not shown). These results indicate that docetaxel treatment in combination with *d*-limonene induces apoptotic cell death in DU-145 prostate cancer cells through mitochondrial dysfunction-dependent manner.

## DISCUSSION

Apoptosis plays an important role in the renewal of the normal prostatic epithelium and in neoplastic prostate, and a reduced apoptosis has been associated with the progression of locally invasive prostate cancers to metastatic disease.[22,23] Because restoring apoptosis has been suggested as a possible therapeutic strategy, a great deal of research has been devoted to understanding the abnormalities in the cellular machinery that causes resistance to apoptosis in prostate cancer cells. Epidemiologic studies, together with extensive basic laboratory findings, support the potential role of phytochemicals, at least as an adjuvant to conventional therapy, in the treatment of prostate cancer.[24,25] Docetaxel, which belongs to the class of chemotherapeutics known as taxanes, is a semisynthetic analog of paclitaxel. Docetaxel's main therapeutic mode of action is the suppression of microtubule dynamic assembly and disassembly, rather than microtubule bundling, leading to apoptosis, and the blocking of Bcl-2 expression.[26,27] Docetaxel plays a major role in the management of advanced HRPC, but when the tumor acquires resistance to docetaxel, an active second-line chemotherapy is needed. For this reason, a broad array of trials that include docetaxel, alone or combined with other drugs are under way.[28–30] *d*-Limonene, one of the common terpenes in nature, has demonstrated low toxicity in humans after single and repeated dosing for up to one year.[31]

In the present study, we have developed docetaxel-based effective combination chemotherapy against advanced prostate cancer using *in vitro* assays. A combination treatment with *d*-limonene sensitized docetaxel-induced cytotoxicity in prostate cancer DU-145 cells at clinically achievable concentrations of both drugs[31] and this combination clearly showed more cytotoxicity to cancer cells in *in vitro* assays compared with normal cells. Cells have an elaborate defense system for protection against free radical-induced damage that involves generation of GSH, cysteine and antioxidant enzymes, including GSH peroxidase, superoxide dismutase and catalase.[32] Further analysis demonstrated H_2_O_2_ as a major cytotoxic molecule of ROS in docetaxel-induced cell death. H_2_O_2_ is known as a cell-death mediator in diverse kinds of cell death including that induced by tumor necrosis factor-α, UV irradiation, and other anticancer drugs.[33–35] Docetaxel alone induced certain amounts of ROS with cell death, whereas addition of *d*-limonene generated a greater amount of ROS, followed by more cell death. At both treated and nontreated phases, the intracellular ROS levels correlated well with the sensitivity to the treatment with docetaxel plus *d*-limonene. The exposure of docetaxel with *d*-limonene makes H_2_O_2_-scavenging systems break down more rapidly, which is typically observed as the decrease of GSH amount. The differential effects of combined treatment on the prostate cancer cells and on normal prostate epithelial cells may be due to the expression of more effective endogenous antioxidant mechanisms in the normal cells than in the malignant ones. It has been reported that docetaxel down regulates several genes for cell proliferation, mitotic spindle formation, transcription factors, and oncogenesis, and up regulates several other genes related to induction of apoptosis and cell cycle arrest in prostate cancer cells, suggesting pleiotropic effects of docetaxel on prostate cancer cells.[36–38]

In this study, we determined that non-toxic low doses of *d*-limonene in the combination treatments with docetaxel have shown a significantly enhanced apoptotic effect on hormone-refractory DU-145 prostate cancer cells. This study showed that combination treatment of prostate cancer cells with docetaxel and *d*-limonene can overcome the resistance of prostate cancer cells to apoptosis and that the generation of ROS, activation of caspase-9 and caspase-3 may play a role in enhanced cytotoxicity. However we did not observe any change in the expression of Fas receptor in the docetaxel alone or docetaxel and *d*-limonene combination treatment in DU-145 cells, which exhibit low Fas expression (data not shown).

## CONCLUSION

Findings presented in this study show, for the first time, that *d*-limonene enhanced the antitumor effect of docetaxel against HRPC *in vitro* without any cytotoxicity to normal prostate epithelial cells. This could be achieved, at least in part, by down regulation of docetaxel-induced expression of Bcl-xL and inducing caspase-mediated apoptosis. These observations warrant further investigation utilizing *in vivo* models that mimic progressive forms of human prostate cancer as well as estimation of pharmacologically achievable doses having biological significance in *in vitro* studies. The positive outcomes of such an *in vivo* study could form a strong basis for the use of *d*-limonene as a novel adjuvant agent to improve docetaxel chemotherapy of human prostate cancer.
